# Immune response induced in mice by a hybrid rPotD-PdT pneumococcal protein

**DOI:** 10.1371/journal.pone.0273017

**Published:** 2022-08-22

**Authors:** Thiago Rojas Converso, Cibelly Goulart, Dunia Rodriguez, Maria Eduarda Souza Guerra, Michelle Darrieux, Luciana C. C. Leite

**Affiliations:** 1 Programa de Pós Graduação Interunidades em Biotecnologia USP-IPT-IB, São Paulo, Brazil; 2 Laboratório de Desenvolvimento de Vacinas, Instituto Butantan, São Paulo, Brazil; 3 Laboratório de Biologia Molecular de Microrganismos, Universidade São Francisco, Bragança Paulista, Brazil; University of South Dakota, UNITED STATES

## Abstract

*Streptococcus pneumoniae* is a human pathogen that colonizes the naso and/or oropharynx and can cause otitis, pneumonia, bacteremia and meningitis. To broaden the protection against pneumococcus, several pneumococcal proteins have been investigated as vaccine candidates. In this study we analyzed the immunological response induced by mouse subcutaneous immunization with a fusion of the Polyamine transport protein D (PotD) and a pneumolysin derivative (PdT), resulting in a hybrid rPotD-PdT protein. Immunization of mice with rPotD-PdT induced increased production of nitric oxide, indicating a higher innate immune response. In agreement, immunization of mice with the hybrid protein was more immunogenic than the individual proteins or their combination, eliciting higher antibody levels. The anti-rPotD-PdT IgG displayed increased binding onto the pneumococcal surface. Furthermore, the anti-rPotD-PdT antisera promoted superior opsonophagocytosis as compared with the other tested formulations. However, despite that the encouraging results *in vitro*, immunization with the hybrid was not sufficient to induce protection against sepsis with a highly virulent pneumococcal strain. taken together, the results suggest that hybrid proteins are an interesting strategy, able to promote improved immune responses, but the inclusion of other antigens may be necessary to promote protection against invasive infections caused by this bacterium.

## Introduction

*Streptococcus pneumoniae* (pneumococcus) is a commensal bacterium that colonizes both naso and oropharynx [1]. Pneumococcus is the major causative agent of bacterial pneumonia, and it may also cause otitis media, meningitis, and bacteremia. The World Health Organization (WHO) estimates approximately 800,000 deaths yearly, mostly of children under 5 and the elderly, as a result of *S*. *pneumoniae* infection [2–5]. A broad-range vaccine could decrease the occurrence of pneumococcal diseases. Currently two types of vaccines are used, but both are limited by the polysaccharide contained in the formulation, presenting restricted coverage. Moreover, several studies have shown the emergence of serotypes not included in the vaccines, an effect known as serotype replacement [4, 6–11]. Due to the cost limitations involved in the implementation of polysaccharide vaccines in many countries, the development of protein-based formulations, which would have lower production costs and potentially broader coverage, emerge as a promising alternative. In particular, different studies have shown that the co-administration of pneumococcal proteins can offer high levels of protection when compared with proteins administered separately [12–16].

The Polyamine Transport Protein D (PotD) belongs to the polyamine transport complex (PotABCD), is located on the bacterial surface [17], and has a binding site for spermidine and putrescine, suggesting that it is the main responsible for capturing these polyamines from the extracellular medium [18]. Some studies have investigated PotD as vaccine candidate in murine models, showing protection in mice against invasive disease and colonization [17, 19–21]. However, despite the promising results in mice, this protein has not been studied in human.

Pneumolysin (Ply) belongs to the family of thiol-activated toxins l [22]; it binds to cholesterol-rich membranes of eukaryotic cells where it undergoes oligomerization leading to the formation of pores which are responsible for the target cell lysis [23]. Ply presents several inflammatory effects and mediates the expression of pro-inflammatory cytokines such as IL-1 β, IL-6 and TNF-α. The instillation of Ply in the lungs of rats reproduced the inflammatory process caused by the bacterium [24, 25]; it has also been shown that Ply could interact with TRL4 with possible adjuvant properties when used in combination with another protein [26, 27]. Ply in its native form presents high toxicity, which prevents its use as a vaccine. PdT is a detoxified form of Ply, generated by site-directed mutagenesis [22, 28]. Thus, other pneumolysoids (detoxified forms of Ply) have completed phase I and II clinical trials with promising results [15, 29].

The fusion of pneumococcal proteins has been used to extend and improve the immune response against pneumococcal proteins alone. Lu *et al*., showed that the fusion of PsaA and PdT elicited antibodies against both proteins [30]; in another study, the fusion of PspA and flagelin was able to improve the protection against invasive challenges [31]. Goulart *et al*., using fusions between PspA and pneumolysoids, showed broadening of the protection induced by PspA against systemic challenge in mice [27]. We have recently shown that a hybrid protein based on PspA and PotD maintained the immunological properties of both parental proteins protecting against invasive disease and reducing colonization [14]. Based on these data and aiming at improving the immune response raised against the PotD protein, this work consisted in constructing a hybrid protein containing PotD and PdT and evaluating the cellular and humoral immune responses induced by its administration in a murine model.

## Materials and methods

### Pneumococcal strains and growth conditions

All pneumococcal strains used in this study are shown in Table 1. Pneumococci were maintained as frozen stocks (-80° C) in Todd-Hewitt broth supplemented with 0.5% yeast extract (THY) and 15% glycerol. Before each experiment, the pneumococci were plated on blood agar and then grown in THY at 37°C in anaerobic conditions.

**Table 1:** *Streptococcus pneumoniae* strains used in this work.

Strain	Serotype	Source	Reference
St 540/99	14	IAL	[14]
St M10	3	UFG	[32]
St 0603	6B	CHHMS	[33]
St A66.1	3	UAB	[30]
St RM200	NE	CHHMS	[33]
St 245/00	14	IAL	[34]
St ATCC6303	3	ATCC	[35]

NE = Non-encapsulated strain.

IAL = Instituto Adolfo Lutz, São Paulo, Brazil.

UFG = Universidade Federal de Goiás, Goiânia, Brazil.

UAB = University of Alabama at Birmingham, AL, USA.

CHHMS = Children’s Hospital, Harvard Medical School, Boston, MA, USA.

ATCC = American Type Culture Collection, Manassas, VA, USA.

### Cloning of *potD* and *potD-pdT* genes

**Fig 1:**
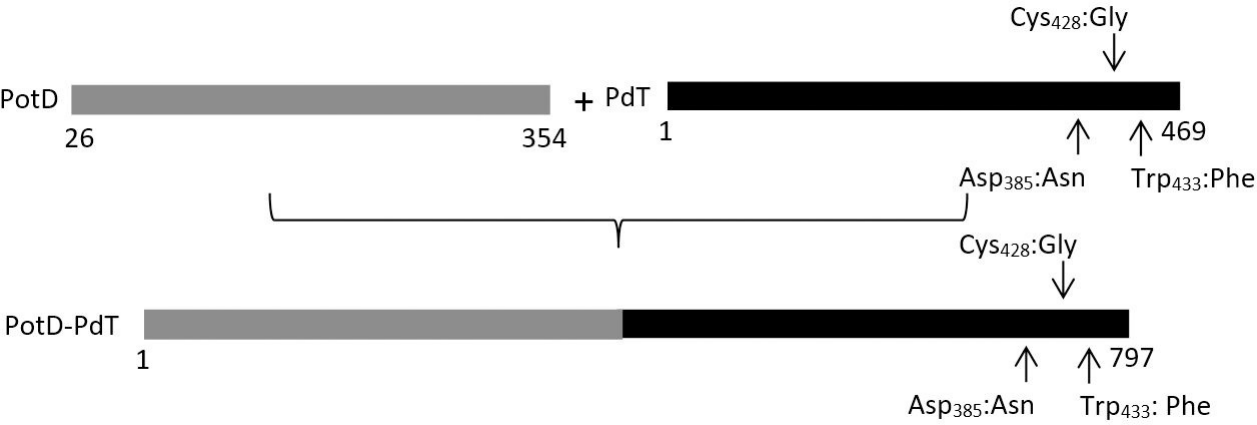
Scheme of recombinant proteins PotD and PdT and assembly of the hybrid. The amino acid substitutions are included for PdT.

The gene fragment corresponding to the mature PotD (without the first 26 amino acids, corresponding to the signal sequence) was PCR-amplified from pneumococcal strain St 540/99. The mutant detoxified pneumolysin gene *pdT* was obtained by PCR from the pQE30-*pdT*, kindly provided by Drs. Richard Malley and James Paton. The primers used to obtain the gene fragments were: PotD F1: 5’ CAT ATG TTA GAT AGT AAA ATC AAT AGT CGA G 3’; PotD R1: 5’ CTC GAG AAG CTT CCG ATA CAT TTT AAA CTG 3’; PdT F1: 5’ AAG CTT ATG GCA AAT AAA GCA GTA A 3’; PdT R1: 5’ CTC GAG CTA ATC ATT TTC TAC CTT ATC C 3’. The *potD* and *pdT* fragments were inserted into pGEM-T easy vector (Promega) and fused through complementary cohesive ends added to the primers, generating the chimeric gene *potD-pdT* (Fig 1), which was further digested with the appropriate restriction endonucleases and ligated to the linearized pQE30 (QIAGEN) expression vector.

### rPotD, rPdT and rPotD-PdT expression and purification

Competent *E*. *coli* M15 (Promega) were transformed with the pQE 30 vector containing the *potD*, *pdT* or *potD-pdT* gene fragments; this vector inserts an N-terminal histidine tag to facilitate the purification. Protein expression was induced in the mid-log-phase cultures by 1 mM IPTG (Sigma). The recombinant proteins were purified from the soluble fraction through affinity chromatography with Ni^2+^ charged chelating sepharose resin (HisTrap Chelating HP; GE HealthCare) in an Äkta Prime (GE HealthCare) apparatus. Elution was performed with 300 mM imidazole.

To remove the lipopolysaccharide (LPS) resulting from the proteins’ production in *E*. *coli*, a wash step was performed consisting in treating 1 mL of purified recombinant proteins (rPotD, rPdt and rPotD-PdT) with 10 μL of TritonX^®^-114 for 30 min at 4°C, followed by incubation at 37°C for 10 min. After centrifugation at 10,600 x g for 10 min at 25°C, the supernatant containing the proteins was removed and transferred to a sterile tube. This washing sequence was repeated 3 times [36]. After three washes, the recombinant proteins were quantified by the Bradford method (BioRad Protein Assay Kit) and stored at -20°C.

### Immunoblotting

The expression and purification of the hybrid protein was confirmed by immunoblotting. Recombinant PotD or PdT (150 ng of each) and 300 ng of the hybrid protein were separated by SDS-PAGE and transferred to nitrocellulose membranes (GE Healthcare). The membrane containing rPotD and the hybrid protein incubated with anti-rPotD, and the membrane containing rPdT and the hybrid protein was incubated with anti- anti-rPdT antisera, both at 1:2,000 dilution, followed by incubation with horseradish peroxidase conjugated goat anti-mouse IgG (diluted 1:2,000; Sigma). Detection was performed with the ECL kit (GE Healthcare).

### Animals and immunization

All animal experiments were approved by the Ethics Committee at Instituto Butantan, São Paulo–SP (CEUAIB), (Protocol Number: 899/12). Female BALB/c mice from Faculdade de Medicina–Universidade de São Paulo (São Paulo, Brazil) were immunized subcutaneously with 3 doses of rPotD (8.4 μg), rPdT (11.6 μg), the co-administered proteins (8.4 μg rPotD + 11.6 μg rPdT) or the hybrid protein, rPotD-PdT (20 μg) at 14 day intervals, using sterile saline solution 0.9% with 100 μg of Al(OH)_3_ as adjuvant. The adjuvant alone in saline was used as control of immunizations. Fourteen days after the last immunization, the animals were bled by retro-orbital puncture and antibody production was evaluated by ELISA.

### Analysis of antibody production in mouse

The presence of specific antibodies in the serum of immunized animals was evaluated by ELISA. Maxisorb plates (Corning) were coated with rPotD or rPdT (1 μg/mL each) and maintained overnight at 4°C; after the incubation, the plates were blocked with 10% skim milk for 30 min, washed 3 times with wash buffer (PBS + 0.05% Tween^®^-20) and incubated with serial dilutions (starting at 1:50 for anti-rPotD and 1:100 for anti-rPdT antibodies) of sera from immunized animals. After the incubation, the plates were washed and incubated with goat anti-mouse IgG (Sigma) at a concentration of 1:10,000 for 1 h. After another wash, the plates were incubated with rabbit anti-goat IgG conjugated with horseradish peroxidase (Sigma) at a concentration of 1:20,000 for 1 h, followed by another six washes. The color reaction was promoted by addition of a substrate solution containing 0.5 mg/ml OPD, 0.0015% H_2_O_2_ in 0.1 M buffer of citric acid/sodium citrate. The reaction was blocked after 10 min by adding 50 μL/well of 4 N H_2_SO_4_. The absorbance was measured at 492 nm, and the antibody concentration was determined using an IgG standard curve.

### Antibody binding assay

Pneumococcal strains were grown in THY to O.D_600_ 0.4–0.5 (corresponding to a concentration of 10^8^ CFU/mL) and aliquots containing 2.5 × 10^6^ CFU (100 μL) were harvested by centrifugation at 1,700 g for 5 min. The pellets were washed with PBS, ressuspended in the same buffer, and incubated in the presence of pooled sera from mice immunized with the hybrid protein rPotD-PdT and the proteins alone or co-administered, at a final concentration of 5% for 30 min at 37°C. Sera from mice that received adjuvant in saline were used as control. After 30 min, the samples were washed with PBS and incubated with 100 μL of PBS containing FITC-conjugated anti-mouse IgG (MP Biomedicals) at 1:100 dilution on ice for 30 min in the absence of light. The bacteria were washed two more times with PBS, ressuspended in 1% paraformaldehyde and analyzed by flow cytometry, using FACS Canto II (BD Biosciences).

### Peritoneal cells

BALB/c mice were injected by intraperitoneal route with 20 μg of Concanavalin A from *Canavalia ensiformis* (ConA, Sigma), euthanized 48 h after treatment and their peritoneal cavities washed with 5 mL of cold PBS. The peritoneal cells were adjusted to 4 × 10^6^ cells/mL in opsonization buffer [37].

### Opsonophagocytic assay

The opsonophagocytic assay using mouse peritoneal cells was performed as described by Goulart, *et al*. [34]. Briefly *S*. *pneumoniae* strains were grown in THY until the concentration of 10^8^ CFU/mL (O.D._600_ 0.4–0.5) and harvested by centrifugation at 1,700 g for 5 min. The pellets were washed once with PBS, resuspended in opsonization buffer [34], and aliquots containing 2.5 × 10^6^ CFU were incubated with heat-inactivated sera from mice immunized with the hybrid protein rPotD-PdT, the proteins alone or co-administered in pool at a final dilution of 1:50 at 37°C for 30 min. The sera had been heat inactivated by incubation at 56°C for 30 min to destroy the activity of serum complement. A pool of sera from sham immunized mice was used as control. After another wash with PBS, the samples were incubated with 10% normal mouse sera (NMS) diluted in opsonization buffer at 37°C for 30 min. The samples were then washed once with PBS and incubated with 4 × 10^5^ peritoneal cells (described on the previous section) composed mainly by macrophages in a MOI of 10:1 (10 bacteria: 1 cell) diluted in opsonization buffer, at 37°C for 30 min with shaking (250 rpm). The reaction was stopped by incubation on ice for 5 min. Ten-fold dilutions of the samples were performed and 10 μL aliquots of each dilution were plated in triplicate on blood agar plates. The plates were incubated overnight at a 37°C for CFU counting.

### Intranasal challenge

The lethal challenge was performed by intranasal route as described by Converso *et al*. [14]. *S*. *pneumoniae* St ATCC6303 were grown in THY medium until the OD_600_ reached 0.4–0.5, aliquoted with THY + 15% glycerol, and kept frozen at −80°C. A suspension containing 3.6 x 10^4^ CFU in 50 μL of sterile PBS was inoculated into one nostril of mice previously anesthetized through the i.p. route with 200 μL of a mixture of 0.5% xylazine and 0.25% ketamine, 7 days after the third immunization. Survival was monitored for 10 days; at the endpoint, all surviving animals were euthanized.

### Statistical analysis

One way ANOVA with a Tukey’s multiple comparison posttest was used for comparison among groups; Student t-test was used for comparison between the control and the immunized group.

## Results

### Production of the hybrid protein rPotD-PdT

**Fig 2:**
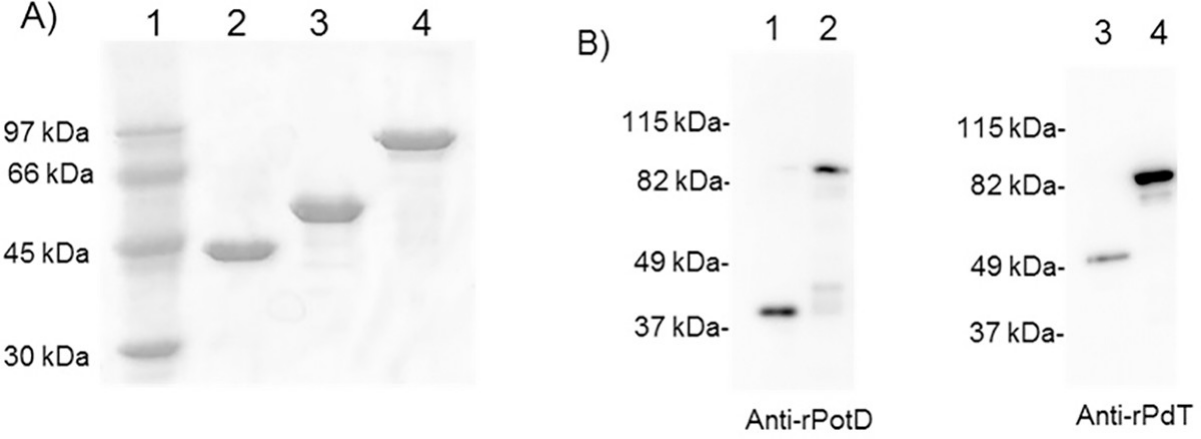
SDS-PAGE and western blot analysis of the purified recombinant proteins. A) SDS-PAGE of the purified recombinant proteins: 1- Molecular marker; 2- rPotD (≈44kDa); 3- rPdT (≈54 kDa); 4- rPotD-PdT (≈96 kDa). B) Western blot of rPotD, rPdT or the hybrid protein. Recombinant proteins were separated by SDS-PAGE and transferred to PVDF membranes, which were incubated with anti-rPotD (left panel) or anti-rPdT (right panel): 1 –rPotD, 2 –rPotD-Pdt and anti-rPdT (right panel): 3 –rPdT, 4 –rPotD-PdT followed by incubation with anti-mouse IgG conjugated with HRP. Detection was performed with an ECL kit (GE Healthcare).

The hybrid and control proteins were expressed in soluble form and purified through nickel affinity chromatography followed by ion-exchange chromatography. At the end of the purification the protein solutions were extracted with Triton X^®^-114 for lipopolysaccharide (LPS) removal [36]. The proteins were separated by SDS-PAGE, revealing low levels of contaminants (Fig 2A). The three recombinant proteins: rPotD, rPdT and rPotD-PdT displayed the expected molecular weights, ≈44 kDa for rPotD [17], ≈54 kDa for rPdT [22] and ≈98 kDa for rPotD-PdT, as predicted by the bioinformatics tool. Western blot analysis (Fig 2B) confirms that the hybrid protein maintained the epitopes of both original proteins, since it was recognized by both anti-rPotD and anti-rPdT sera.

### Mouse immunization with the rPotD-PdT hybrid induces high levels of specific antibody levels

**Fig 3:**
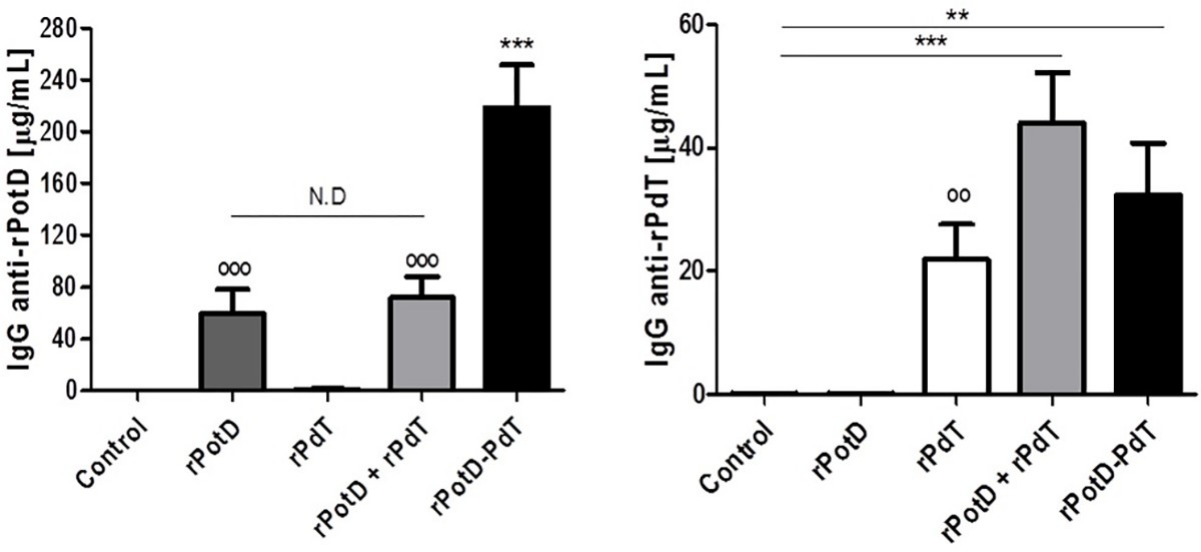
Antibody production induced by immunization. Sera were collected 14 days after the third immunization with rPotD, rPdt, rPotD + rPdT and rPotD-PdT, and individually tested against rPotD or rPdT. Serum from animals that received Aluminum hydroxide diluted in saline was used as control. Results are the mean ± SD of 5 mice. *** p<0.001 and ** p<0.01, for multiple comparison by ANOVA and°°° p<0.001 and°° p<0.01, for paired analysis between the immunized and control groups (t-test). N.D = Not Different.

To investigate the humoral immune response elicited by the administration of rPotD-PdT, mice were immunized with the hybrid protein or the respective control proteins, bled by retro-orbital puncture, and the serum separated and used for evaluation of anti-rPotD or anti-rPdT total IgG by ELISA (Fig 3). Groups immunized with rPotD or the co-administered proteins had comparable levels of anti-rPotD antibodies; immunization with the hybrid protein increased ~2.5 –fold the level of anti-rPotD antibodies (Fig 3, left panel). The groups immunized with either rPdT, the co-administered proteins or the hybrid protein produced comparable levels of anti-rPdT IgG (Fig 3, right panel).

### Antibody binding onto the surface of different pneumococcal strains

**Fig 4:**
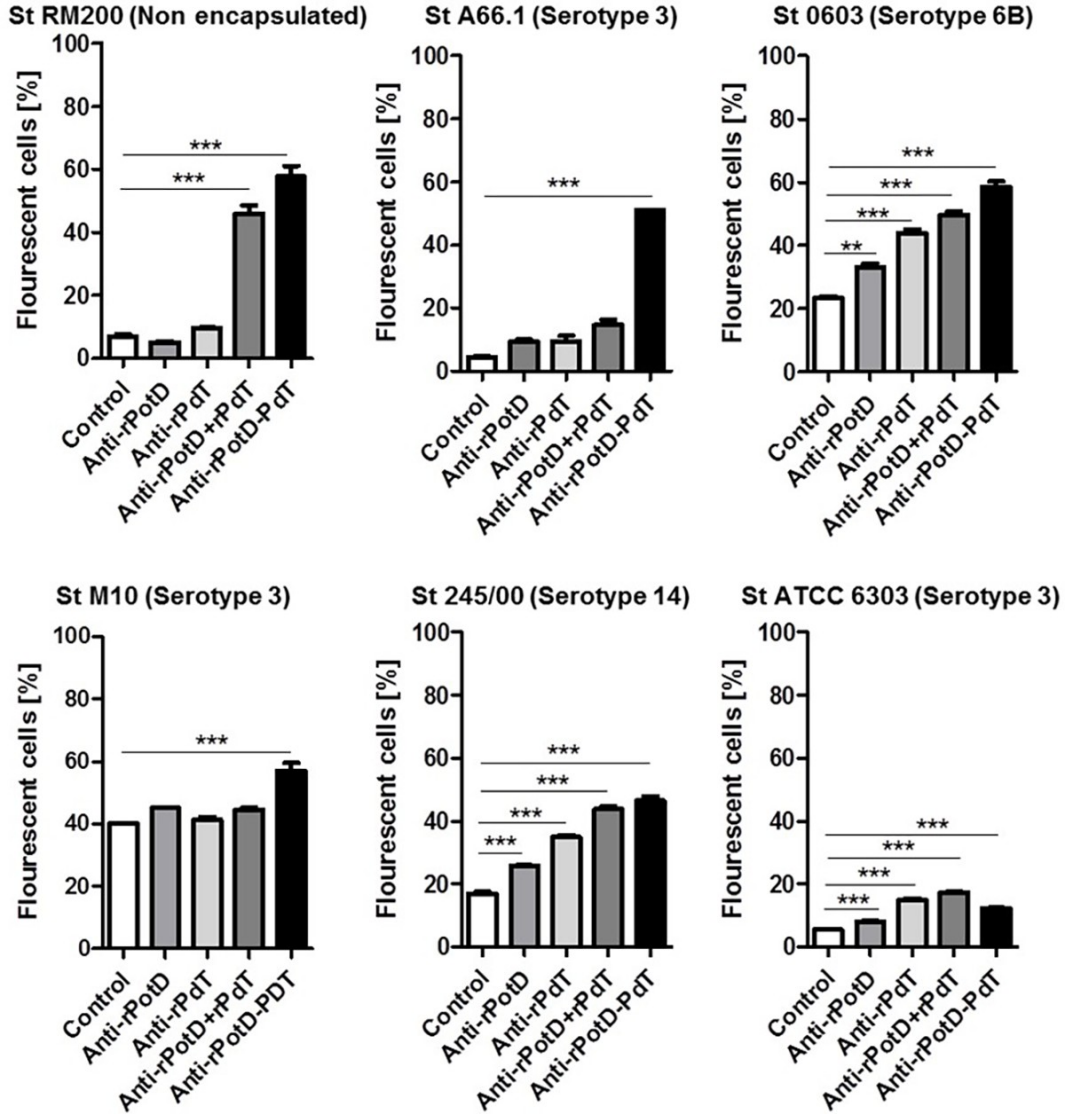
Antibody binding to the pneumococcal surface. Pneumococcal strains St RM200, St A66.1, St 0603, St M10, St 245/00 and St ATCC6303 were incubated with anti-sera from mice immunized with rPotD, rPdT, rPotD + rPdT, rPotD-PdT or the adjuvant alone in saline (control), followed by incubation with anti-IgG mouse conjugated with FITC and analyzed by FACS. The percentage of fluorescent bacteria (10^1^ fluorescence intensity units) was calculated for each sample. *** p <0.001; ** p <0.01; * p <0.05. The values correspond to the mean + SD of five serum samples for each group.

The binding assay was performed to investigate the functional activity of the antibodies recognition and binding to the native proteins on the surface of different pneumococci. Five encapsulated strains and the non-encapsulated, autolysin-negative, St RM200 strain (which also carries a substitution of Ply for PdT) were evaluated. The percentage of positive cells after incubation with specific antisera is shown for each bacterium (Fig 4); cells with fluorescence intensity higher than 10^1^ were considered positive.

Antibodies induced against the rPotD-PdT showed an increased capacity to bind to the bacterial surface of all pneumococcal strains tested when compared to the control serum. For the non-encapsulated RM200 strain, both the antisera from mice immunized with the hybrid and the co-administered proteins showed increased binding. Sera from all immunized groups (rPotD, rPdT, the combination or the hybrid) displayed increased binding to strains St 0603, St 245/00 and St ATCC 6303 (serotypes 6B, 14 and 3, respectively). Interestingly, only the antisera from the hybrid rPotD-PdT showed increased binding to the serotype 3 strains, St.A66.1 and St M10. Therefore, binding of the anti-hybrid antiserum was comparable or superior to each protein alone or the combination in all pneumococci. In contrast, antibodies to the combination rPotD+rPdT or the isolated proteins recognized only three of the five strains.

It is important to mention that although detectable binding was observed for all strains, there were marked variations in the percentages of positive cells among the strains. For the St ATCC6303 strain, binding was not superior to 20% of total cells for any of the tested sera, suggesting an inefficient recognition. For the other strains, maximum binding ranged from 50 to 60% of total cell counts.

### *In vitro* opsonophagocytosis mediated by anti-rPotD-PdT antisera and a complement source

**Fig 5:**
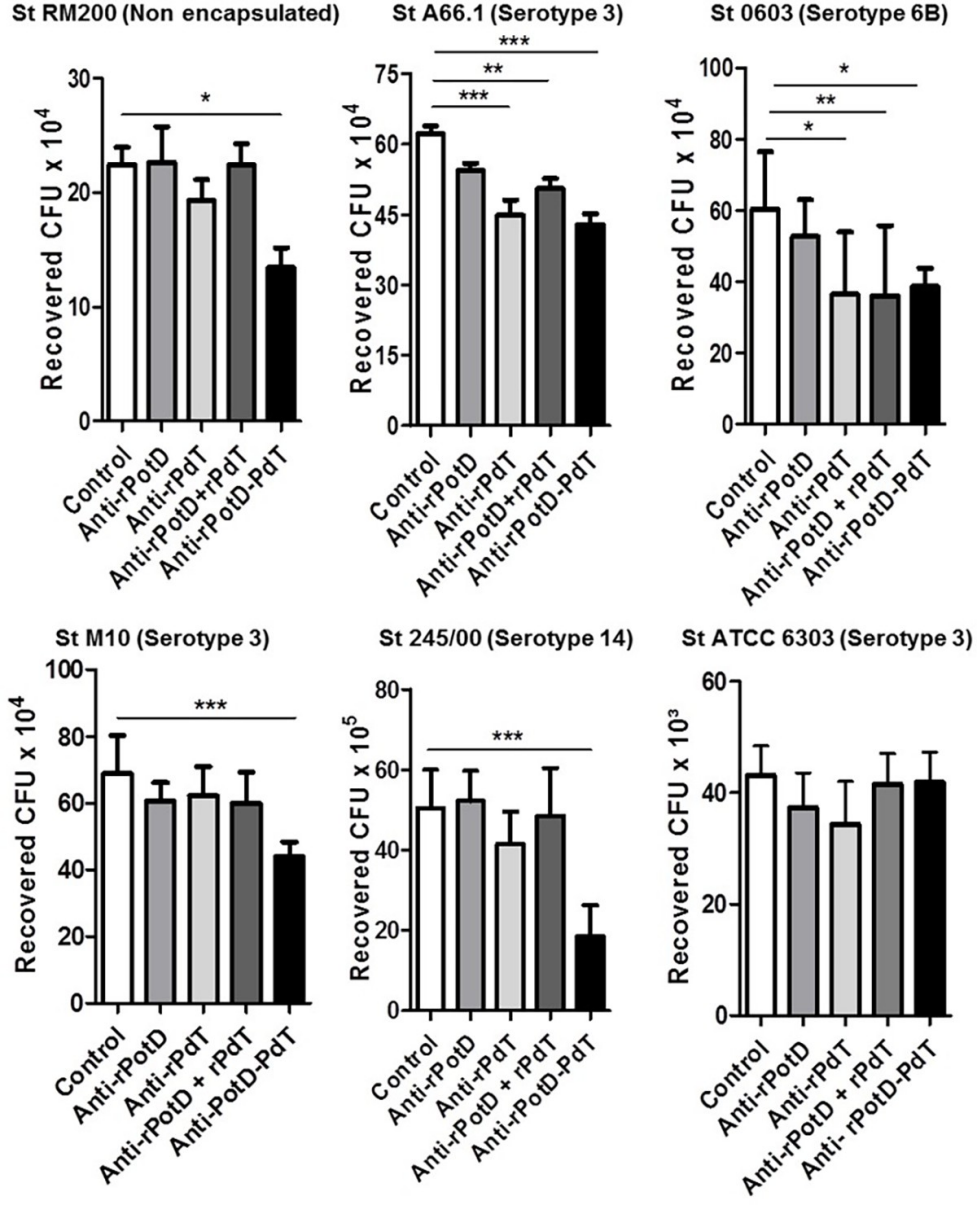
Pneumococcal phagocytosis mediated by specific antibodies in the presence of complement. Pneumococcal strains St RM200, St A66.1, St 0603, St M10, St 245/00 and St ATCC6303 were incubated with antisera from mice immunized with rPotD, rPdT, rPotD + rPdT or rPotD-PdT and NMS as complement source, followed by incubation with mouse peritoneal phagocytes and plated on blood agar plates. CFU recovered were counted after 18 h. * p<0.05; ** p<0.01; *** p<0.001 for treated versus control or between immunized groups, as indicated. The values correspond to mean + SD of three independent experiments.

The ability of the sera to enhance opsonophagocytosis of pneumococci *in vitro* was evaluated using murine peritoneal cells. These cells were incubated with the bacteria in the presence of specific antisera and a source of complement. The result is expressed by the number of Colony Forming Units (CFU) recovered. It can be observed that only incubation of bacteria with the anti-rPotD-PdT serum significantly reduces the number of recovered CFUs from strains St RM200, St M10 and St 245/00, whereas antisera from all the control groups did not (Fig 5). For strains St 0603 and St A66.1, there was a reduction in the number of recovered CFUs when incubated with anti-rPdT, anti-rPotD+rPdT and anti-rPotD-PdT sera as compared with the control group. None of the tested sera reduced the number of recovered CFUs of the St ATCC6303 strain in comparison with the control group. These results suggest that antibodies generated by the immunization with the hybrid protein rPotD-PdT somehow induce antibodies capable of eliciting a more efficient immune response than the proteins alone or co-administered.

### Intranasal lethal challenge

**Fig 6:**
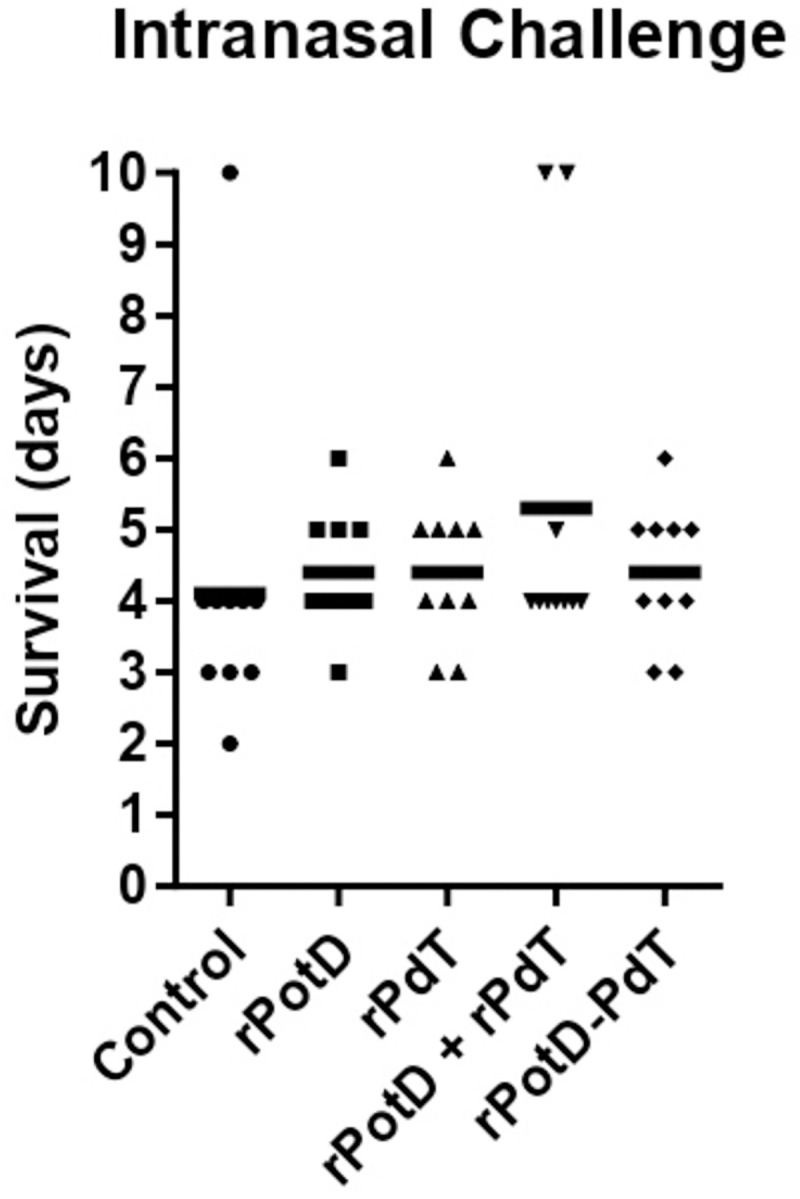
Survival times (days) for mice after intranasal challenge. Groups of 10 BALB/c mice were immunized subcutaneously with the indicated antigens and challenged intranasally 7 days after the third immunization with St ATCC6303 (3.6 × 104 CFU). Each dot represents one mouse. The horizontal lines denote the median survival time for each group. Control group is composed by animals injected with adjuvant in saline only.

To test the protective potential of the vaccine candidate rPotD-PdT, 7 days after the last immunization, the animals were intranasally challenged with the highly virulent pneumococcal strain St ATCC6303. Fig 6 shows the survival of each animal after challenge. Despite the correlates of protection observed in all tests performed in this work, the immunization with rPotD-PdT was not able to confer protection against the tested strain.

## Discussion

Some studies have shown that the fusion of pneumococcal proteins can result in expanded and improved immune responses with encouraging results [14, 16, 27, 30]. The pneumolysoid, PdT, was chosen for fusion with PotD because it is a widely studied protein and its combination or fusion with other proteins has been shown to improve the respective immune responses [27, 30]. Other pneumolysoids are also being evaluated in clinical trials [15, 29]; PdT, on the other hand, is being evaluated in phase 2 clinical trials as part of a whole cell vaccine where Ply was substituted for PdT (ClinicalTrials.gov Identifier: NCT01537185). The aim of this work was to characterize the immune response induced by mouse immunization with a hybrid protein comprising PotD in fusion with PdT, in comparison with each protein alone or the combination (rPotD + rPdT). All proteins were expressed in high levels in the soluble fraction of *E*. *coli*, and purification was successful, with very low contaminant levels. The immunoblotting analysis revealed that the hybrid protein was recognized by anti-rPotD and anti-rPdT antibodies, indicating that the construct maintained epitopes of both original proteins. A similar result has been described for other vaccines based on pneumococcal fusion proteins [14, 27, 38].

Since the hybrid protein induced the production of antibodies against both proteins, in quantities that were comparable or higher than the recombinant proteins alone or in combination, it is possible to infer that no antigenic competition occurred between rPotD and rPdT in this chimeric molecule. These findings are in agreement with previous studies where the fusion of pneumococcal proteins led to an increase in antibody production against each vaccine component [14, 27, 30, 31]. Furthermore, the increased production of anti-rPotD antibodies induced by the hybrid in comparison to the groups receiving rPotD alone or in combination with rPdT suggests a possible adjuvant effect by rPdT in the fusion [26].

Previous studies have shown that anti-rPotD antibodies can bind to bacterial serotypes with thin or no capsule [17, 39]. In the present work, antibodies generated against rPotD were shown to bind to serotype 14, 6B and one type 3 strain. However, the overall binding of anti-rPotD observed was low when compared to previous reports [39]. Knowing that PotD is accessible to antibody binding and since the anti-rPotD levels and the fluorescence intensity in our study were similar to previous reports [14, 17, 39], one possible explanation for the lower binding capacity may be the difference in the genetic background of the tested strains. Variations in capsular serotype, and possible interactions with other surface proteins could interfere with PotD exposure and consequently, with its accessibility to antibodies. [17, 19, 21, 39]. Shah *et al*., showed that native PotD is more expressed in bacteria growing *in vivo* than *in vitro* [39]; this could explain the lower binding capacity observed.

An enhancement in antibody binding was observed in all strains following incubation with antibodies to the hybrid rPotD-PdT, being the only one able to confer this level of binding. This improved binding has been reported previously for PspA-Pneumolysoid fusions, and correlated to an enhanced protection against invasive pneumococcal challenge [27, 30]. The exact mechanism by which the fusion of pneumococcal proteins results in an improved immune response is yet unknown. One possible explanation is that fusion of rPotD and rPdT could alter their structure and lead to exposure of epitopes that influence the quality of the generated antibodies.

In accordance with the binding results, antibodies induced against the hybrid protein increased phagocytosis of five out of the six strains tested, including different serotypes. Since this effect was not observed for rPotD alone, one can infer that rPdT can act as an adjuvant, augmenting the immune response to rPotD in the fusion [26]. In fact, this adjuvant effect by fusion has been demonstrated previously for PdT or flagellin in fusion with other pneumococcal proteins [27, 30, 31]. The ability of the induced antibodies to recognize diverse pneumococci and to promote an increase in bacterial clearance by phagocytosis are strong indicators of the protective potential of rPotD-PdT vaccine formulation.

Lastly, we investigated the ability of this vaccine formulation to confer protection against lethal pneumococcal infection. Despite the encouraging results in the *in vitro* assays that supported our idea that a fusion between rPotD and rPdT could lead to an improved immune response against pneumococcal infection, no protection was observed with any of the formulations tested. Few papers have shown that rPotD is protective in animal models; in these works the protection was evaluated by utilization of another mouse strain: CBA/n (which do not make immune responses to polysaccharides) [17, 19], or by a different administration route: intranasal immunization [21]. These differences in immunization and challenge route, as well as the mouse strain, may account for the differences in protection in those studies. Still, our results are in agreement with our previous observation where PotD alone was not able to protect against invasive challenge [14]. Despite that, we believed that the combination between PotD and PdT could lead to protection.

On a whole, the results suggest that a vaccine formulation containing rPotD in fusion with rPdT, promotes improved immune responses against the bacterium as compared with the administration of the isolated proteins alone or in combination, especially the opsonophagocytic properties of the antibodies produced. However, the responses elicited by vaccination were not enough to protect against sepsis by a highly virulent strain. Therefore, the inclusion of other pneumococcal antigens may increase the protective potential of this formulation.

## Supporting information

S1 Raw images (PDF)
