# Supplementary material for: Immune response induced in mice by a hybrid rPotD-PdT pneumococcal protein
Source: PLoS One. 2022 Aug 22;17(8):e0273017. doi: 10.1371/journal.pone.0273017 (PMC9394809; doi:10.1371/journal.pone.0273017)
Supplement: S1 Raw images — (PDF) [file pone.0273017.s001.pdf]

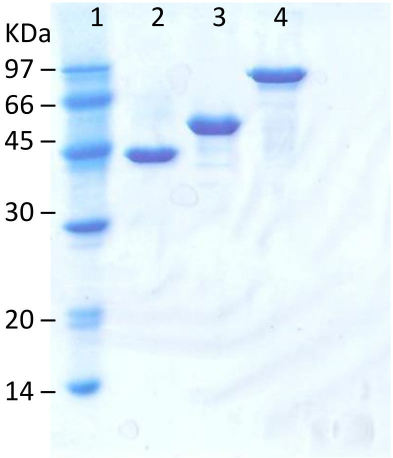

Polyacrylamide gel is the uncropped, original 12% polyacrylamide gel, the molecular weight is shown on lane 1. Lane 2 rPotD; lane 3 – rPdT and lane 4 – rPotD-PdT.

Western Blot anti-rPotD

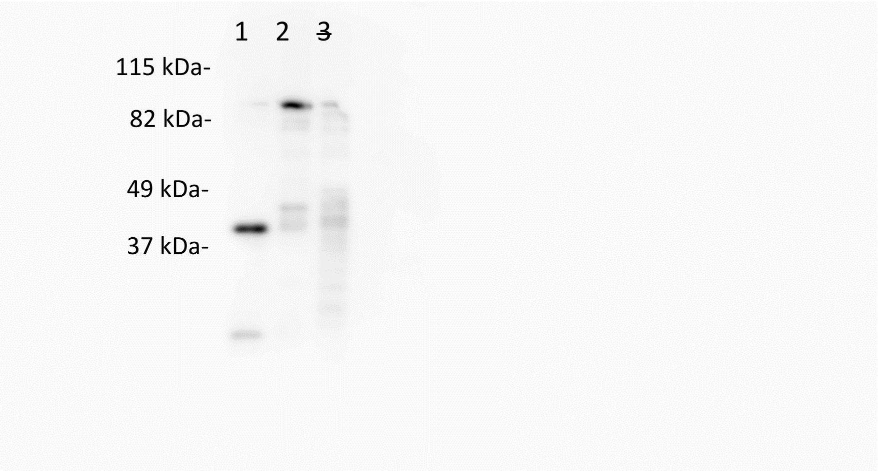

This Wester blotting gel is the uncropped, original Wester blot using anti-rPotD antibodies against 1 rPotD, 2- rPotD-PdT (protein used in the study) and 3 – rPotD-PdT (different purification method). Lane number 3 was not used in the manuscript being removed from the manuscripts figure.

Western Blot anti-rPdt

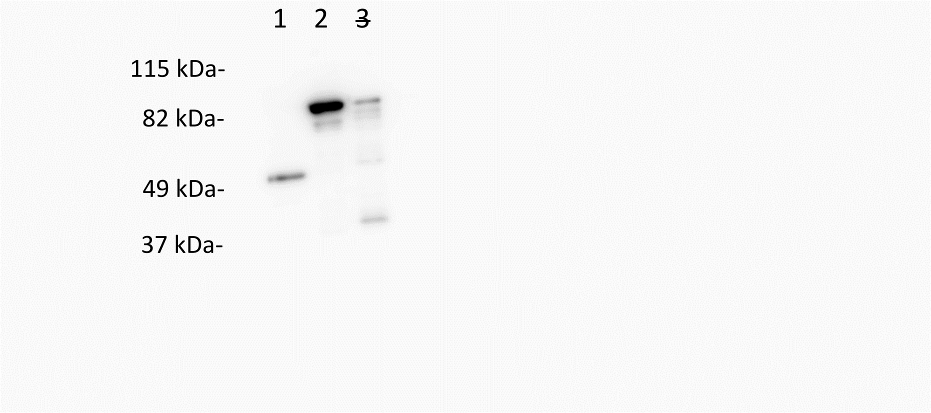

This Wester blotting gel is the uncropped, original, Wester blot using anti-rPdT antibodies against 1 rPdT, 2- rPotD-PdT (protein used in the study) and 3 – rPotD-PdT (different purification method). Lane number 3 was not used in the manuscript being removed from the manuscript's figure.
